# Car–Parrinello molecular dynamics of QMAP-DMSO microsolvation: first-shell structure and solvent-induced polarization

**DOI:** 10.1007/s00894-026-06920-3

**Published:** 2026-08-29

**Authors:** Renato Medeiros, Francisco A. P. Osório, Clodoaldo Valverde, Ademir J. Camargo

**Affiliations:** 1 Laboratory of Applied Molecular Modeling and Simulation (LaMMAS), State University of Goiás, Anápolis, Goiás Brazil; 2 Institute of Physics, Federal University of Goiás, Goiânia, Goiás Brazil; 3 Paulista University — UNIP, Goiânia, Goiás Brazil; 4 Theoretical and Structural Chemistry of Anápolis, (QTEA) State University of Goiás, Anápolis, Goiás Brazil

**Keywords:** Car–Parrinello molecular dynamics, First-shell microsolvation, Solvent-induced polarization, Dipole autocorrelation, Orientational correlation, QMAP-DMSO

## Abstract

**Context:**

Directional solute–solvent contacts can influence the electrostatic response of donor–acceptor chromophores, although such local effects are often represented only as averaged contributions in continuum solvent models. Quinolinylaminophenol (QMAP) in dimethyl sulfoxide (DMSO) was examined as an explicit first-shell microsolvation model to clarify how local solvent organization couples to QMAP conformation and dipolar response. The Car–Parrinello molecular dynamics trajectory indicates a geometric reorganization around 12–13 ps, accompanied by changes in the first-shell DMSO dipolar arrangement. Within the limitations of the finite microsolvated model, these results suggest that transient first-shell solvent organization contributes to the modulation of the local electrostatic environment of QMAP, rather than acting only as a static dielectric background.

**Methods:**

The gas-phase QMAP geometry was optimized at the CAM-B3LYP/6–311 + + G(d,p) level using Gaussian 16. The explicit QMAP–DMSO model contained one QMAP molecule and 21 DMSO molecules in a periodic cubic cell and was propagated by Car–Parrinello molecular dynamics using the CPMD code. The production trajectory was obtained with the PBE exchange–correlation functional, Troullier–Martins norm-conserving pseudopotentials, a plane-wave basis set, NVT equilibration at 300 K, and a restarted thermostatted CPMD production protocol, with a total analyzed trajectory length of approximately 32 ps. Structural, hydrogen-bond, dipole-moment, IR-like, and orientational-correlation analyses were carried out using in-house Python scripts.

## Introduction

**Fig. 1:**
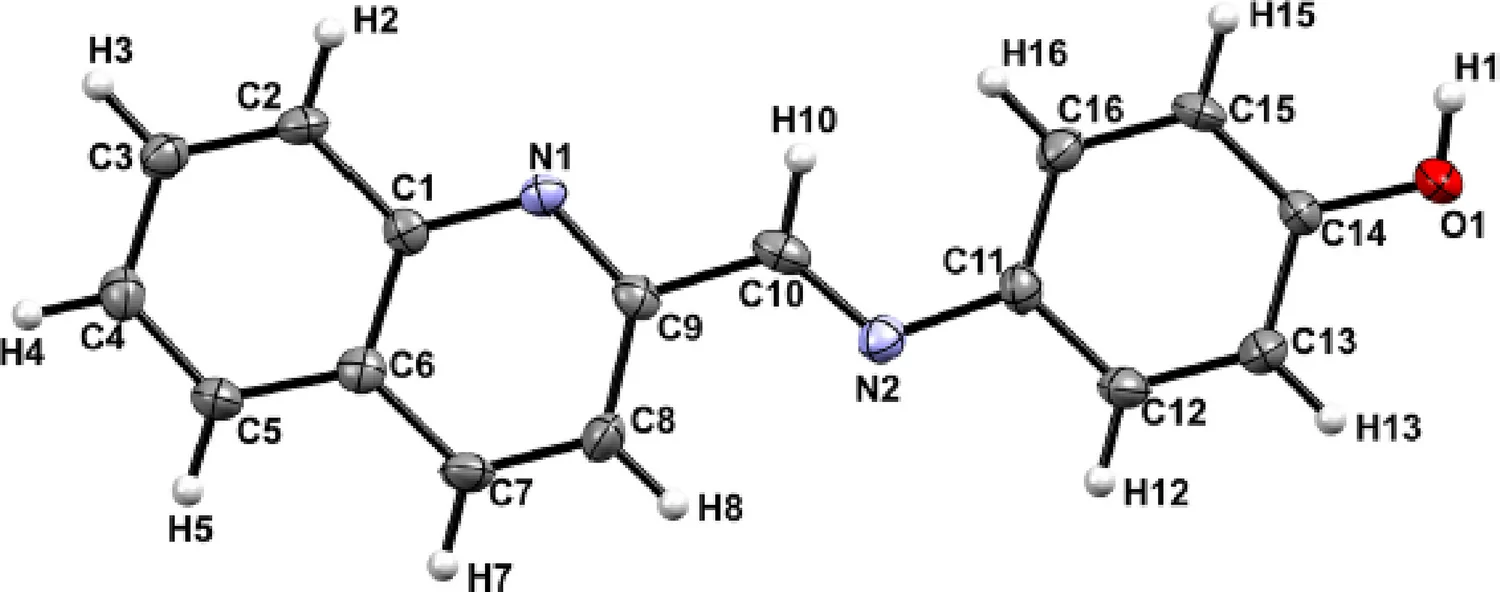
Molecular structure of 4-(quinolin-2-ylmethylene)aminophenol (QMAP) with the atomic numbering used in the analysis

Donor–acceptor organic chromophores have been studied because conformational and environmental changes can alter the charge distribution, the dipole moment, and the optical response. Quinoline phenol derivatives, such as 4-(quinolin-2-ylmethylene)aminophenol (QMAP, Fig. 1), are suitable systems for this type of analysis. In these systems, the quinoline fragment, the imine bridge, and the donor phenolic group form a conjugated structure whose electronic response depends on the intramolecular geometry and local solvation [1–3]. In this context, QMAP is an appropriate molecular model to examine how explicit solvent molecules influence polarization in the first solvation shell.

Continuum solvent models describe the average dielectric stabilization efficiently. However, they do not explicitly represent the directionality, intermittency, and spatial selectivity of short-range contacts between solute and solvent. In DMSO, a strongly polar aprotic solvent and efficient hydrogen-bond acceptor, the relevant local field is expected to depend on the orientation of individual DMSO molecules around the phenolic and heteroaromatic regions of QMAP. Car–Parrinello molecular dynamics (CPMD) [4] can be applied to this problem because the electronic structure and the nuclear motion evolve in a coupled manner. This allows the analysis of the dipolar response from a Car–Parrinello trajectory.

This work addresses a specific local question regarding how an explicit first shell of DMSO reorganizes around QMAP and how this reorganization relates to the QMAP conformation and solvent-induced polarization. The system is therefore treated as an ab initio microsolvation model, rather than a simulation of DMSO in the liquid phase. Thus, the study was limited to discussing the local structure of the first solvation shell, without aiming to obtain converged macroscopic transport, dielectric, or spectroscopic properties of liquid DMSO.

## Computational details

The isolated QMAP geometry was first optimized in the gas phase at the CAM-B3LYP/6–311 + + G(d,p) level with Gaussian 16 [5, 6]. The CAM-B3LYP functional was chosen because it is suitable for the study of donor–acceptor systems, especially when long-range charge redistribution may be relevant. The diffuse and polarization functions of the basis set contribute to the description of the phenolic OH group and the imine and quinoline nitrogens at the same level of treatment. The optimized structure was used as the initial solute geometry for the explicit microsolvation model. Additional gas-phase and PCM(DMSO) optimizations followed by frequency calculations were performed with both CAM-B3LYP and PBE. All four structures were true minima. The solvent-induced geometrical change was negligible: the heavy-atom RMSD between gas-phase and PCM structures was 0.0119 Å with CAM-B3LYP and 0.0138 Å with PBE, and the maximum heavy-atom displacement remained below 0.024 Å. PCM stabilized QMAP by approximately 7.6–7.9 kcal mol^−1^ and increased the calculated dipole moment, indicating appreciable electronic polarization without an important conformational change. Therefore, use of the gas-phase starting geometry is not expected to materially affect the reported first-shell structural trends, although it may influence the initial dipole moment and the earliest stage of relaxation.

The microsolvated system was constructed by placing QMAP in a periodic cubic cell and surrounding it with twenty-one DMSO molecules. This number of solvent molecules was selected to build an explicit first-shell microsolvation environment around the accessible molecular surface of QMAP while keeping the ab initio molecular dynamics simulation computationally feasible. The model was therefore not designed to reproduce bulk liquid DMSO, but to provide an atomistic description of the local solvent environment directly interacting with the chromophore. Consequently, it should be interpreted using short-range descriptors, such as contact distances, local dipolar organization, and direct hydrogen-bond motifs, and not as a converged representation of DMSO in the liquid phase.

The explicit microsolvated model was composed of one QMAP molecule and 21 DMSO molecules, totaling 241 atoms, in a neutral periodic cubic cell with a side length of 16.0 Å. Before the molecular dynamics run, the electronic wavefunction was optimized at fixed nuclear geometry using the ODIIS procedure, with an orbital convergence threshold of 1.0 × 10^−7^. The Car–Parrinello molecular dynamics simulations were then performed with the CPMD code, following the Car–Parrinello formalism [4], using the PBE exchange–correlation functional [7] and Troullier-Martins norm-conserving pseudopotentials [8] for H, C, N, O, and S. A plane-wave kinetic-energy cutoff of 25 Ry was used together with a dual-grid parameter of 8. No symmetry was imposed during the simulation. Test optimizations showed that the heavy-atom RMSD between CAM-B3LYP and PBE geometries was 0.0588 Å in the gas phase and 0.0656 Å in PCM (DMSO), with no change of conformational minimum and no imaginary frequencies. The maximum difference in heavy-atom internal distances was approximately 0.11–0.12 Å. Thus, the functional change may affect absolute geometrical parameters, dipole moments, and local interaction strengths, but the structural differences are modest and are not expected to alter the qualitative trajectory-based conclusions. Accordingly, the CPMD results are interpreted mainly in terms of relative structural and dipolar changes along the trajectory and qualitative coupling between QMAP conformation and first-shell DMSO organization.

The equilibration stage was carried out by restarting from the optimized wavefunction and coordinates. A time step of 5.0 atomic units, corresponding to approximately 0.121 fs, was employed. The ionic degrees of freedom were coupled to a massive Nosé-Hoover thermostat at 300 K [9], while the fictitious electronic degrees of freedom were also thermostatted to preserve the stability of the Car–Parrinello propagation. The equilibration input consisted of 1000 steps. The subsequent production trajectory was generated from restarted wavefunction, coordinates, velocities, Nosé variables, and accumulated quantities. Each production segment contained 100,000 steps, corresponding to approximately 12.1 ps, and the analyzed trajectory was obtained by concatenating the restarted production segments. Atomic coordinates were sampled every five molecular dynamics steps, while restart information was written frequently to allow stable continuation of the trajectory.

Because the simulation contains a finite number of explicit DMSO molecules in a periodic microsolvated cell, the model was interpreted as a first-shell solvation environment rather than as a converged representation of bulk liquid DMSO. Therefore, the structural and dipolar analyses were restricted to local descriptors, such as solute–solvent contact distances, hydrogen-bond motifs, internal QMAP coordinates, and dipole fluctuations of the finite QMAP-DMSO assembly.

Trajectory analysis was carried out with in-house Python scripts [10]. VMD [11] was used only for visual inspection and verification of the trajectory and molecular configurations. The descriptors were chosen to connect molecular structure and electrostatic response: internal QMAP coordinates describing the conjugated backbone, the molecular dipole of QMAP extracted frame by frame, the total dipole of the QMAP-DMSO cluster, pair-distance distributions involving the phenolic and heteroatom regions, donor–acceptor hydrogen bonds defined by simultaneous distance and angular criteria, and orientational-correlation functions of the cluster dipole. For non-correlated dipole statistics, frames were subsampled at intervals of at least 200 fs to reduce autocorrelation bias. To improve reproducibility, the Python scripts used in the analysis, together with processed input/output files needed to reproduce the figures, are provided in a public repository: https://github.com/ProfRenatoMedeirosFisico/qmap-cpmd-analysis.

The interpretation considers two main limitations. First, the finite cell with 21 DMSO molecules represents an explicit first-shell microsolvation cluster, not a converged liquid. Second, the 32-ps trajectory allows the identification of consistent qualitative mechanistic correlations, but it is too short to quantitatively describe IR band shapes, rotational diffusion, or long-range dielectric relaxation. All spectral and orientational analyses are therefore reported as qualitative descriptors of the microsolvated cluster.

## Results and discussion

### Internal geometry and solvent-induced conformational transition

**Fig. 2:**
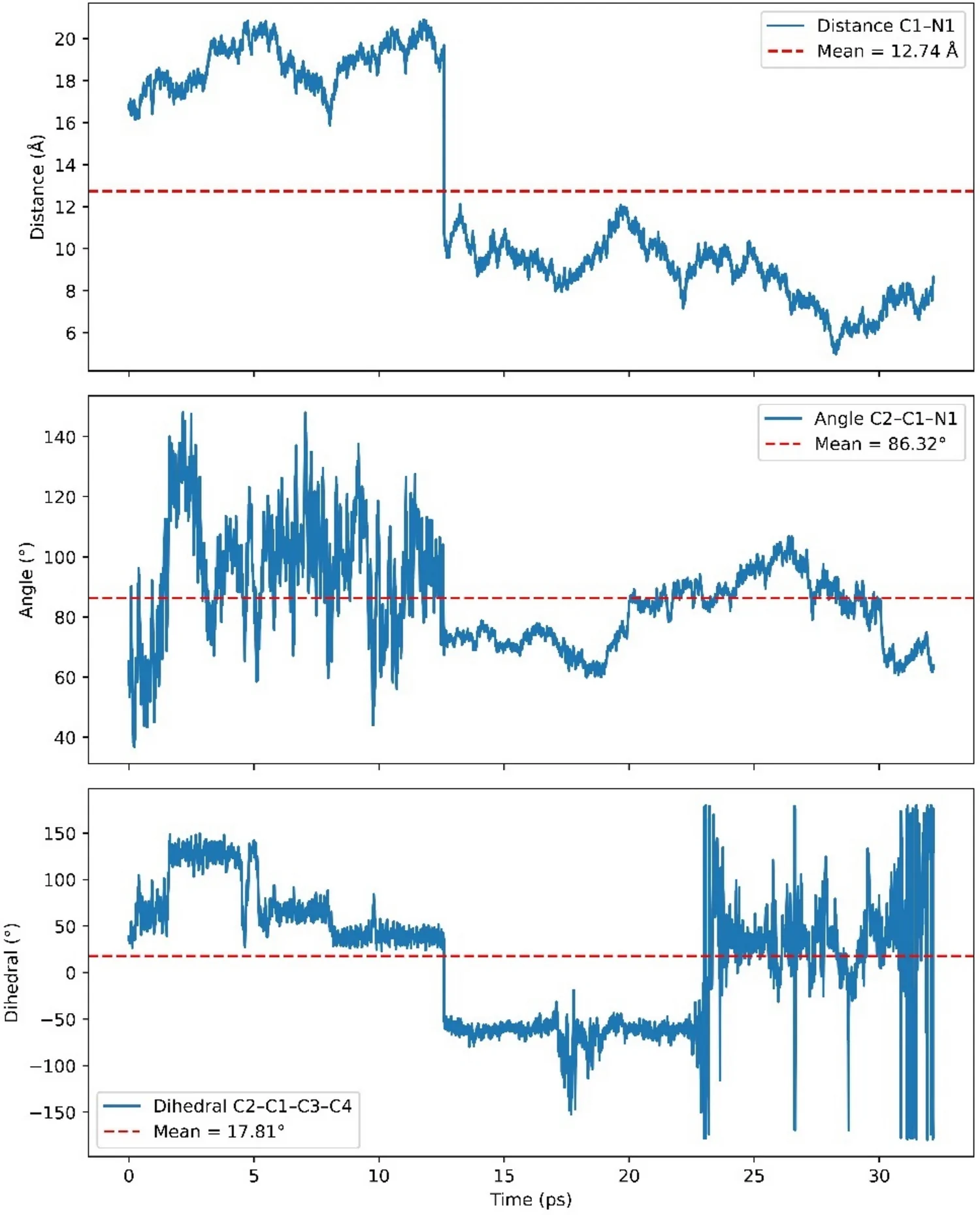
Time evolution of QMAP internal parameters: (top) $\mathrm{C}1-\mathrm{N}1$ distance, (middle) $\mathrm{C}2-\mathrm{C}1-\mathrm{N}1$ angle, and (bottom) $\mathrm{C}2-\mathrm{C}1-\mathrm{C}3-\mathrm{C}4$ dihedral. Red dashed lines show the global mean of each descriptor over the full trajectory

Figure 2 summarizes the trajectory-resolved internal geometry of QMAP. The monitored descriptors track the relative position of key heteroatoms, the opening of the molecular framework, and the torsional state of the conjugated backbone. The simultaneous variation of these descriptors around 12 to 13 ps provides the structural reference point for the mechanistic interpretation discussed below.

The traces of the internal coordinates indicate two distinguishable regimes, separated by a sharp change near 12.5 ps. Before this point, QMAP samples a broader conformational region; after it, the angular and torsional descriptors settle into a different range and the backbone explores a new local geometry. Because one distance descriptor is sensitive to periodic-boundary unwrapping, the absolute pre- and post-transition values are not used as standalone structural parameters. The most reliable observation is the synchronous change between independent descriptors.

This rearrangement, accompanied by the solvent, should be considered because it relates geometry and electrostatic response. A purely static description of solvation would tend to represent only an average stabilization of QMAP. However, the trajectory indicates a discrete conformational event associated with a change in dipolar behavior. Thus, the transition is treated as a mechanistic indication of coupling between the solute backbone and its first DMSO shell.

### Molecular dipole of QMAP and total dipole of the cluster

**Fig. 3:**
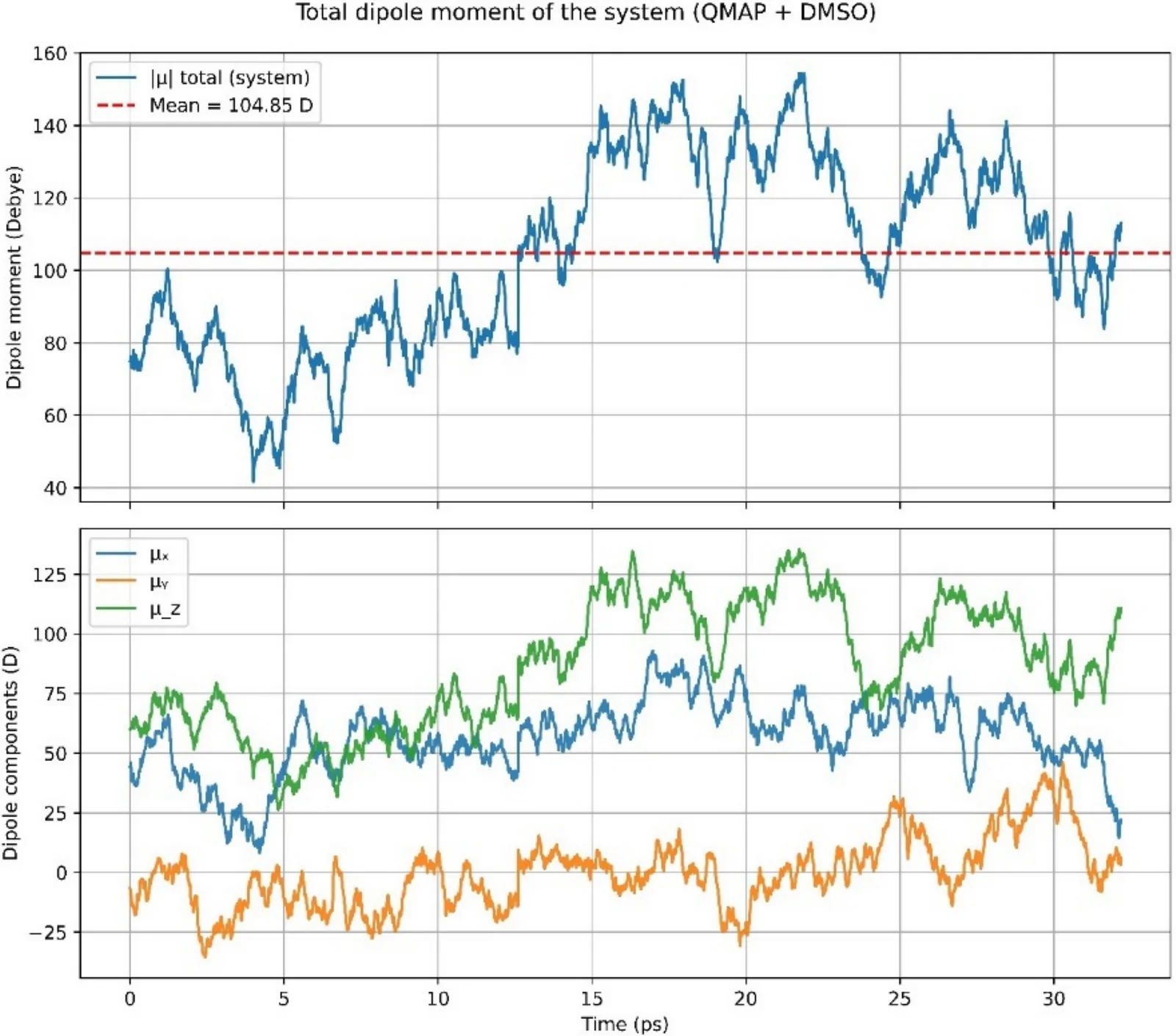
Total dipole moment of the QMAP + DMSO cluster: (top) magnitude |μ| and (bottom) Cartesian components μx, μy, μz

**Fig. 4:**
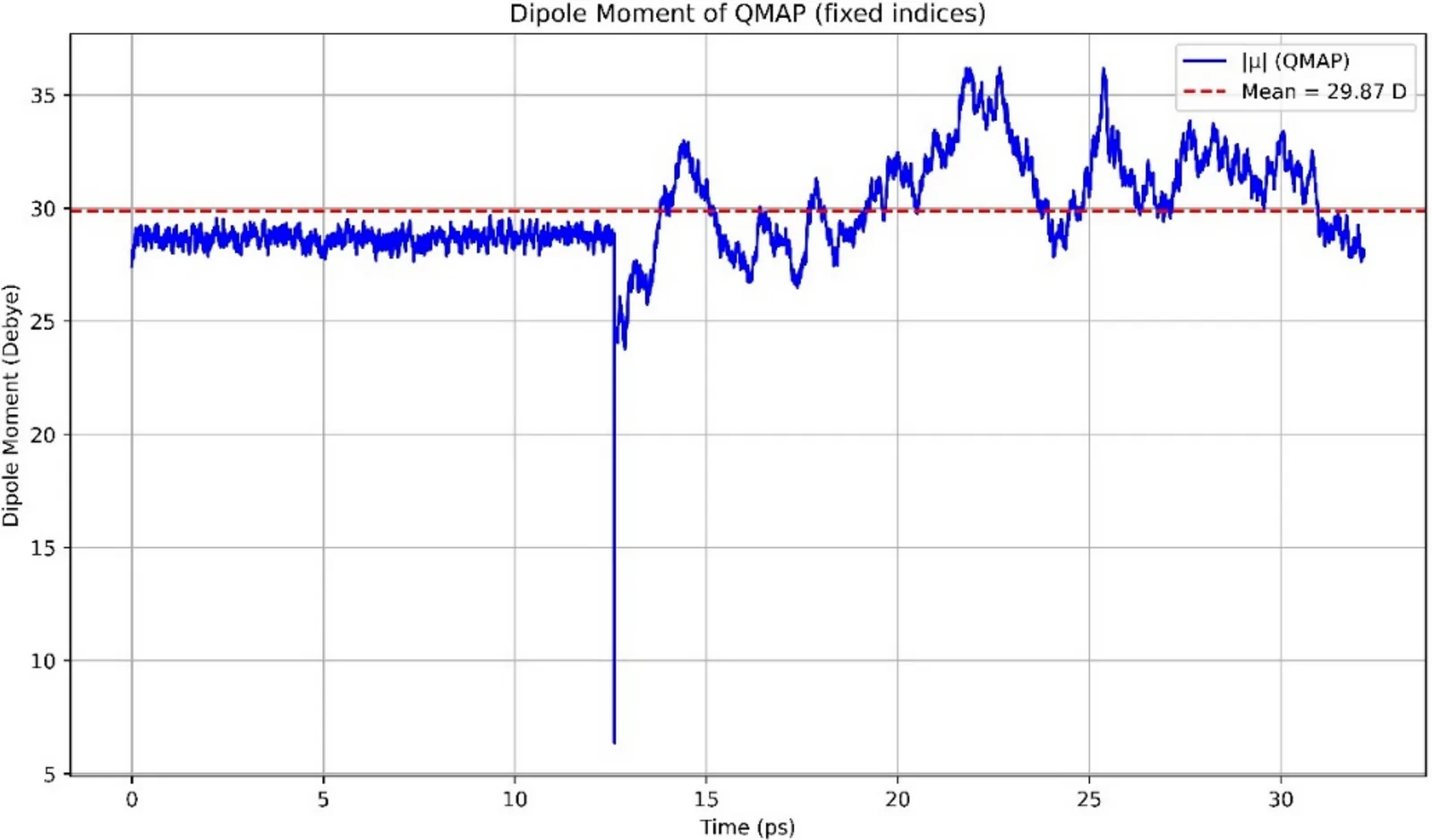
Time evolution of the molecular dipole magnitude of QMAP extracted from the explicit-solvent trajectory. Dashed line indicates the global mean, 29.87 D

Figures 3 and 4 present the total dipole of the QMAP-DMSO cluster and the QMAP-only dipole magnitude, respectively. This comparison distinguishes the moderate dipolar variation of the solute from the collective dipolar response of the finite microsolvated assembly.

The QMAP molecular dipole remains close to 30 D on average, but its plateau and variance change after the conformational transition. The isolated downward spike near 12.6 ps is not interpreted as a physical depolarization event because it coincides with the coordinate-unwrapping sensitivity already identified in the structural analysis. The most reliable trend is the shift from a narrower dipole regime before the transition to a broader regime after the transition.

The total cluster dipole is much larger than the QMAP-only dipole and is dominated by the collective orientation of the 21 DMSO molecules. The change observed after the conformational transition suggests that solvent reorganization contributes to the total electrostatic response more strongly than the intrinsic dipolar variation of the isolated QMAP. The preferential Cartesian component observed in Fig. 3 is a property of the finite periodic cluster and should not be read as macroscopic polarization of a bulk liquid.

The main result of the dipole analysis is mechanistic in nature. The explicit first DMSO shell changes its collective dipolar organization around QMAP, modifying the finite-cluster dipole, while the QMAP molecular dipole shows a more moderate variation. This distinction is relevant for the solvent-induced polarization model proposed in the “Molecular mechanism of solvent-induced polarization” section.

### Pair distance distributions around QMAP

**Fig. 5:**
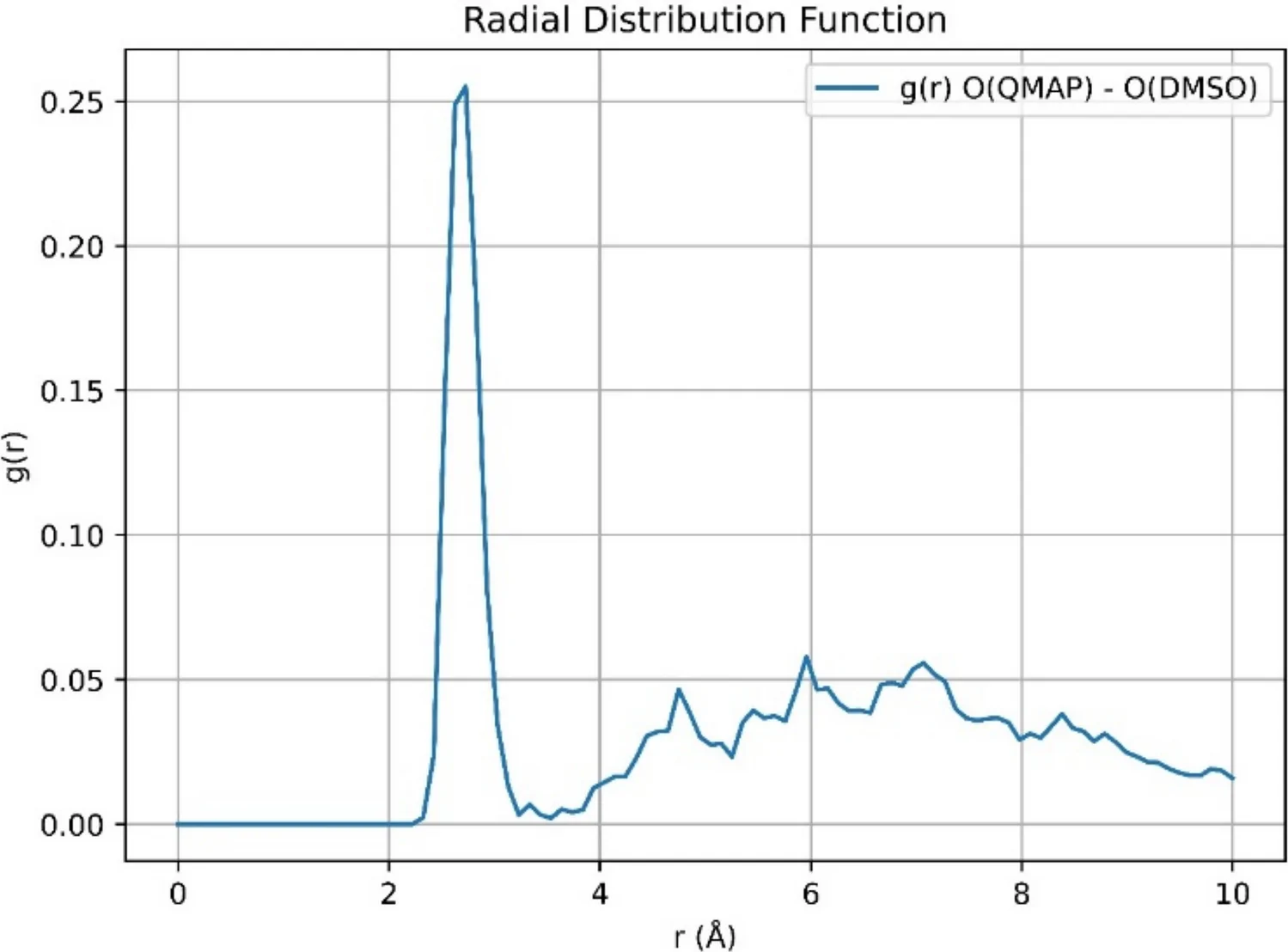
O(QMAP)-O(DMSO) pair distance distribution along the trajectory. A single sharp feature is observed near 2.7 Å, followed by a broad low-amplitude region between 4 and 8 Å

**Fig. 6:**
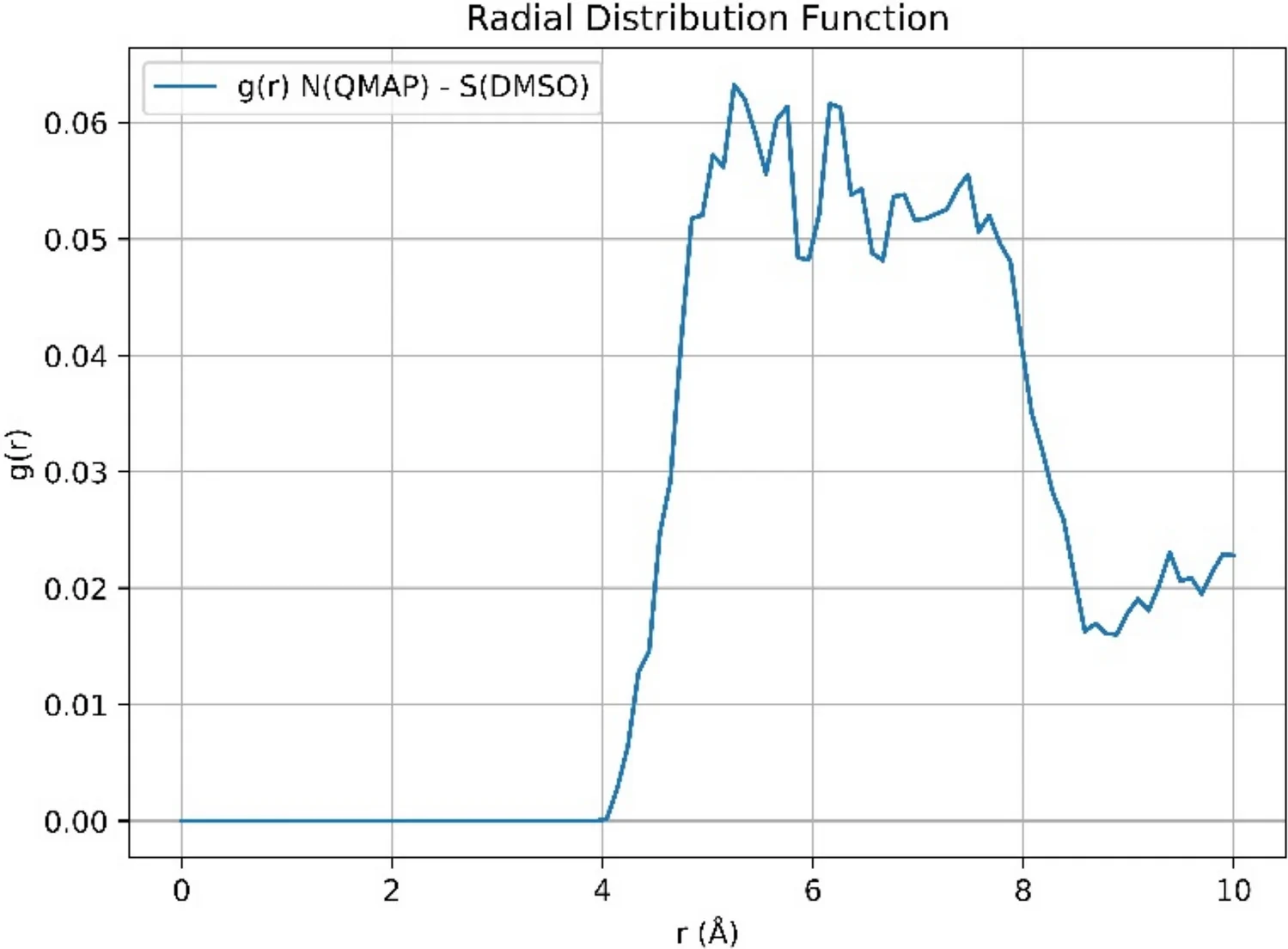
N(QMAP)-S(DMSO) pair distance distribution along the trajectory. No first shell is resolved below 4 Å; the curve rises to a broad plateau between 5 and 8 Å

Figures 5 and 6 report pair-distance distributions selected to probe first-shell organization around the chemically distinct regions of QMAP. In a bulk simulation, these functions would normally be normalized as radial distribution functions, but in the present finite microsolvation cluster they are used only as contact-density descriptors. These distributions are used to identify which regions of the solute are most frequently approached by DMSO molecules within the sampled first shell.

The O(QMAP)-O(DMSO) distribution shows a short-range feature near 2.7 Å, indicating selective approach of DMSO to the oxygen-containing region of QMAP. This contact distance is consistent with an arrangement involving the phenolic group. However, the distance alone is not sufficient to assign a hydrogen bond. Directional criteria are therefore required, and those criteria are examined explicitly in the “Hydrogen-bond analysis” section.

The complementary distribution involving the imine/quinoline nitrogen region and DMSO sulfur does not show an equivalent short-range first-shell feature. This contrast suggests that the DMSO organization around QMAP is chemically selective. The phenolic region concentrates the main local contact motif, while the nitrogen-containing region contributes mainly to a more diffuse spatial occupation in the cluster.

Together, the pair-distance distributions indicate that the explicit first shell is not isotropic around QMAP. The DMSO preferentially samples the phenolic side of the molecule, providing the local structural indications for the solvent-induced polarization mechanism.

### Hydrogen-bond analysis

**Fig. 7:**
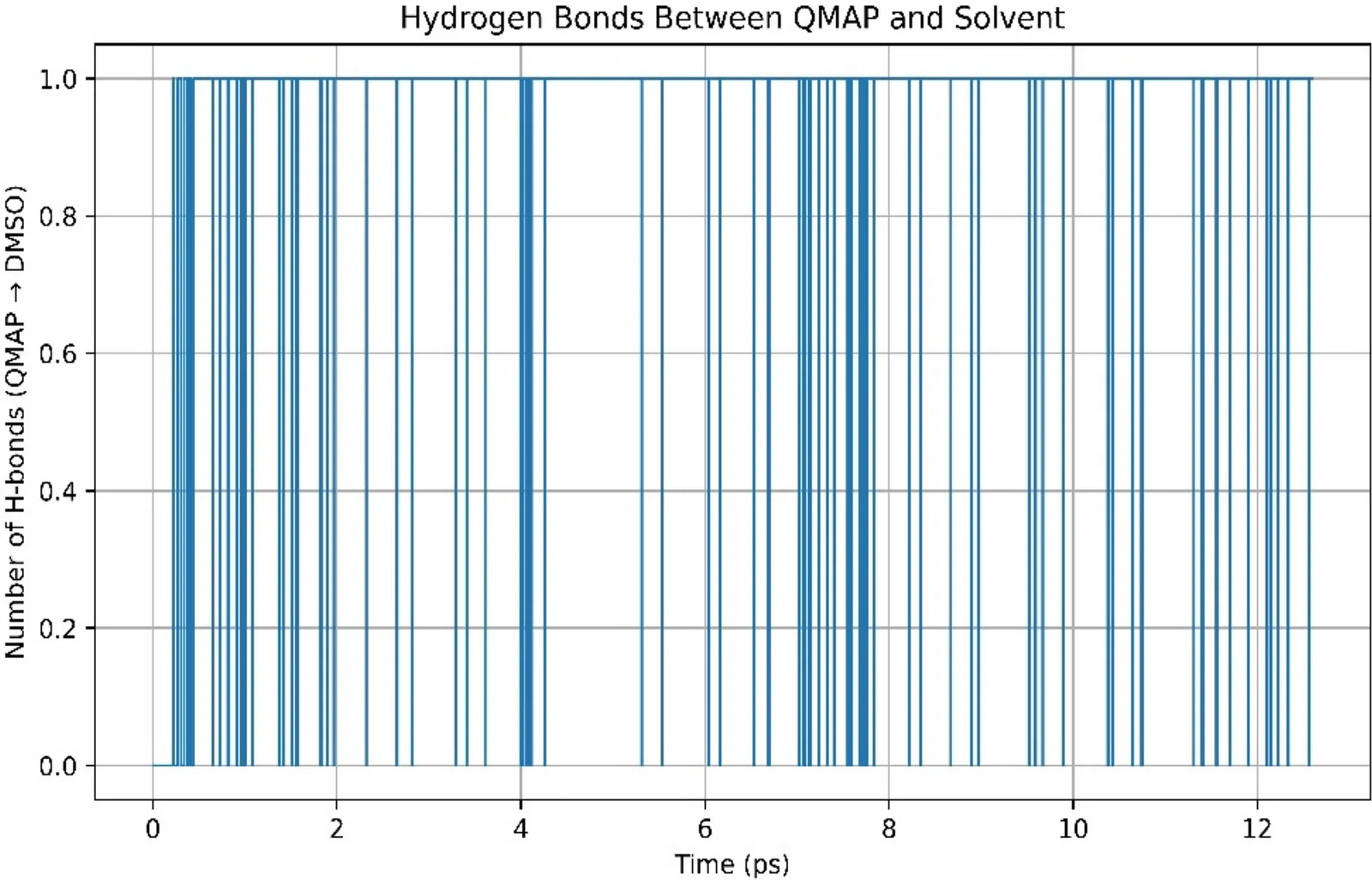
Number of donor–acceptor hydrogen bonds between QMAP and DMSO (H···A ≤ 2.5 Å, D-H···A ≥ 150°) during the first 12.5 ps of the trajectory

Figure 7 evaluates the directional hydrogen bonds between QMAP and DMSO during the interval prior to the transition. The analysis is limited to this window because the subsequent coordinate reorganization complicates a uniform full-trajectory lifetime assignment. The purpose is therefore not to obtain a converged H-bond lifetime, but to test whether the short O(QMAP)-O(DMSO) contact identified in Fig. 5 has directional hydrogen-bond character.

The hydrogen-bond count oscillates between 0 and 1, showing that the interaction is intermittent rather than persistent. DMSO therefore acts as a transient acceptor for the phenolic OH group, not as the center of a stable hydrogen-bond network. This result is chemically important: the polarization mechanism is driven by repeated local alignment and reorientation of first-shell DMSO molecules, not by permanent bond formation.

### Molecular mechanism of solvent-induced polarization

The trajectory supports a mechanistic solvent-induced polarization model in which QMAP conformational rearrangement and first-shell DMSO organization are correlated. The event near 12–13 ps is interpreted as a trajectory-dependent local reorganization of the solute backbone and the surrounding solvent field. As the conjugated scaffold changes geometry, the electrostatic environment sampled by nearby DMSO molecules changes, and the DMSO dipoles respond by reorganizing around the phenolic region.

The resulting feedback can be summarized as follows: QMAP conformation modulates the local charge distribution, first-shell DMSO molecules reorganize around the new electrostatic environment, and the reorganized solvent field modifies the dipole of the microsolvated cluster. Because the relevant contacts are transient, this modulation is fluctuation-driven rather than dependent on a persistent hydrogen-bond network.

This model has a molecular-design implication that should be tested in future work. Substitution at or near the phenolic site may alter hydroxyl acidity, steric accessibility, and local DMSO alignment, thereby changing both the magnitude and variability of the dipolar response. The contribution of this modeling study is to establish a chemically interpretable relationship between the three-dimensional structure of the first solvation shell and solvent-induced polarization in the microsolvated model.

### Qualitative IR spectrum from the dipole time series

The qualitative IR-like spectrum was estimated from the autocorrelation function of the total cluster dipole. The dipole autocorrelation function was defined as

$${C}_{\mu \mu }\left(t\right)=\langle \overrightarrow{\mu }(0)\cdot \overrightarrow{\mu }(t)\rangle$$

where the dipole vector refers to the full QMAP-DMSO cluster and the brackets denote an average over time origins within the available trajectory. The spectral intensity was then approximated by the frequency-weighted Fourier transform of this correlation function [12],

$$I\left(\omega \right)\propto {\omega }^{2}{\int}_{0}^{T}{C}_{\mu \mu }\left(t\right){e}^{-i\omega t}dt$$

**Fig. 8:**
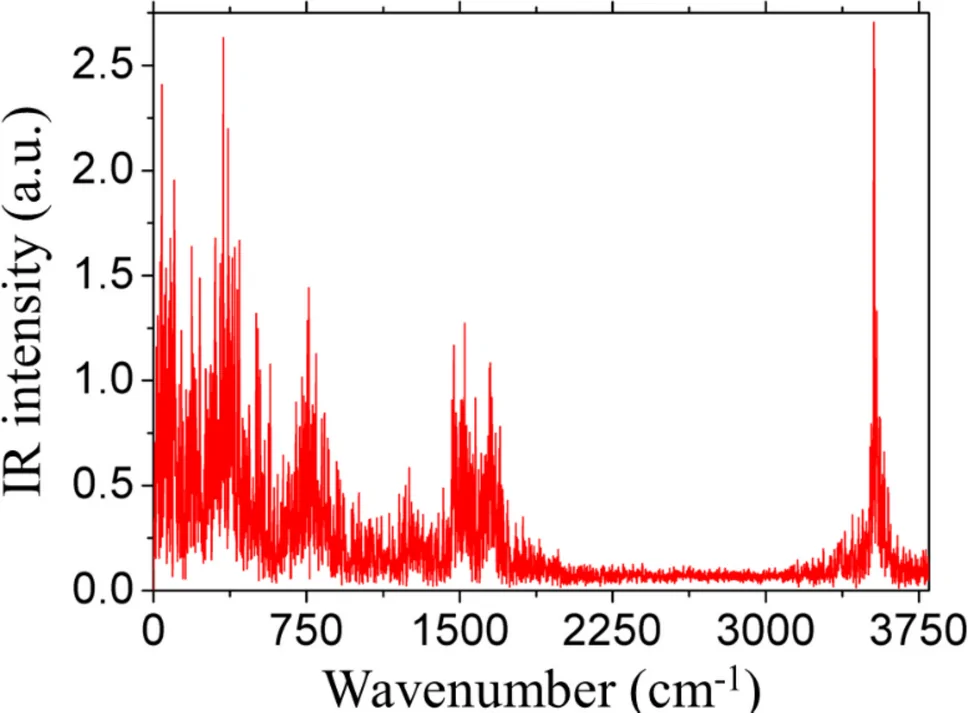
Qualitative IR intensity computed from the ω^2^-weighted Fourier transform of the total dipole autocorrelation function over the trajectory

This expression was used only as a qualitative descriptor of dipole-active motions in the finite microsolvated cluster. Because the trajectory is approximately 32 ps long and contains only 21 DMSO molecules, the calculated profile should not be interpreted as a quantitatively converged infrared spectrum of bulk DMSO solution. The resulting qualitative IR-like spectrum is shown in Fig. 8.

The resulting profile separates low-frequency collective/librational motion from higher frequency intramolecular activity of the solute. The 0–300 cm ^-1^ cluster motions, whereas the 300–1500 cm^-1^ region is dominated by soft interval contains the expected fingerprint activity of an aromatic donor–acceptor system. Features between 1500 and 1800 cm^-1^ are consistent with coupled imine and ring-stretching motions. The high-frequency region contains weak C-H activity and a tentative phenolic O-H contribution. The narrowness and exact positions of these features reflect the finite trajectory length and absence of instrumental broadening, so the spectrum is used as supporting evidence for dipole-active local motions rather than as a direct experimental prediction.

### Exploratory orientational relaxation of the cluster dipole

Orientational relaxation of the finite cluster was inspected through normalized correlation functions of the unit dipole vector,

$$\widehat{\mu }\left(t\right)=\frac{\overrightarrow{\mu }(t)}{\left|\overrightarrow{\mu }(t)\right|}$$

**Fig. 9:**
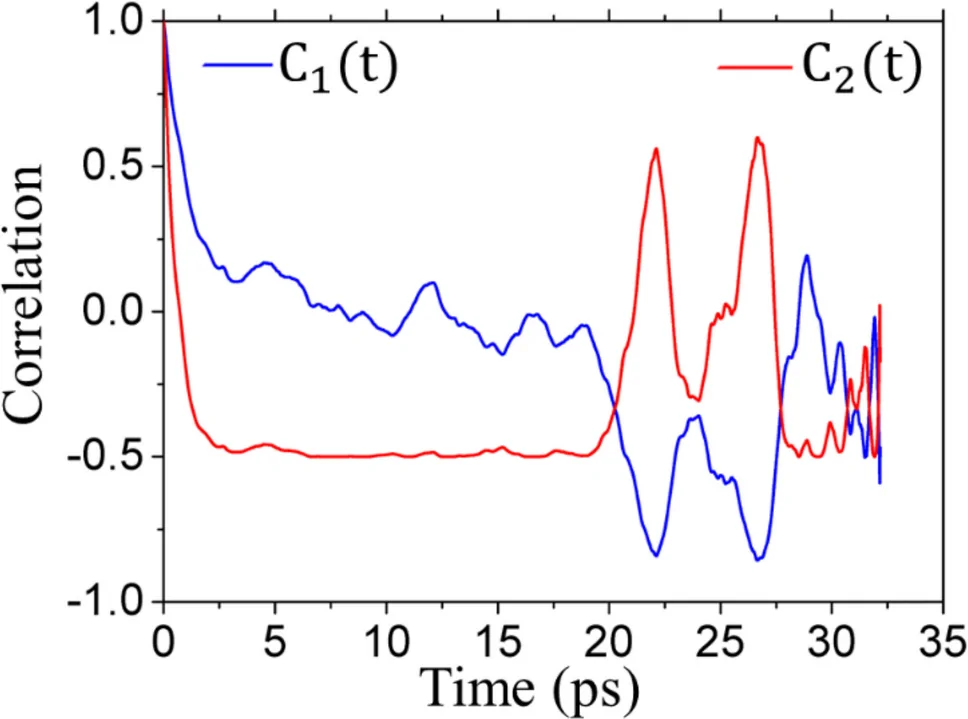
Orientational correlation functions of the unit dipole of the full cluster: *C*₁(*t*) (first rank) and *C*₂(*t*) (second rank)

The general rank -l orientational correlation function was written as

$$C_l(t)=\langle P_l[\hat{\mu}(0)\cdot\hat{\mu}(t)]\rangle$$

where ${P}_{l}$ is the Legendre polynomial of rank l. The first- and second-rank functions plotted in Fig. 9 were therefore

$$C_1(t)=\langle \hat{\mu}(0)\cdot\hat{\mu}(t)\rangle$$

$$C_2(t)=\langle \frac{3[\hat{\mu}(0)\cdot\hat{\mu}(t)]^2-1}{2}\rangle$$

An apparent correlation time was estimated only as an order-of-magnitude descriptor by integrating the first-rank function up to a cutoff time ${t}_{c}$ before the noise-dominated tail,

$${\tau}_{1}\left({t}_{c}\right)={\int}_{0}^{{t}_{c}}{C}_{1}\left(t\right)dt$$

This treatment is exploratory because the finite cluster is anisotropic, the sampled trajectory is short, and the long-time tail is sensitive to the integration cutoff.

The first-rank function decays rapidly during the first picoseconds, consistent with librational motion and partial reorientation of the total cluster dipole inside the instantaneous solvent cage. The second-rank function loses correlation even faster and enters a noisy long-time regime, as expected for a short trajectory of a small, anisotropic cluster. Integrating C_1_(*t*) only up to the stable part of the decay gives an apparent relaxation time on the picosecond scale. This value is reported as a qualitative measure of cluster-dipole decorrelation, not as a Debye relaxation time for bulk DMSO. For comparison, bulk dielectric and orientational relaxation are commonly discussed in terms of dipolar correlations, Kirkwood-type factors, and rotational-relaxation models [13–15]. In the present microsolvation model, the first- and second-rank correlation functions should not be interpreted as signatures of separate near-solute and far-from-solute solvent populations, as may be considered in some bulk-solvent analyses. Both C_1_(*t*) and C_2_(*t*) are calculated from the same unit vector of the total cluster dipole and correspond to different Legendre ranks of the same collective orientational dynamics. They are therefore interpreted as complementary descriptors of angular decorrelation within the finite QMAP-DMSO cluster, rather than as evidence for distinct solvent regimes.

## Scope and limitations of the model

The scope of the model is intentionally limited. The system is an explicit first-shell microsolvation cluster and not a converged bulk liquid; the IR-like spectrum and orientational correlation functions are qualitative cluster descriptors; and the 32 ps trajectory is too short for quantitative transport, dielectric relaxation, or fully converged band-shape analysis. The conclusions are therefore based on internally consistent mechanistic correlations among structure, contact motifs, and dipole response rather than on macroscopic solvent observables. In a larger bulk-like system, the first-shell directional contacts identified here would be expected to remain relevant, especially around the phenolic region of QMAP, but additional solvent shells would introduce long-range dielectric screening, broader solvent-orientation distributions, and slower collective fluctuations. Consequently, the absolute dipole moment of the total simulated system would not be directly comparable to that of the present finite cluster, and the IR-like response would probably show stronger broadening, frequency shifts, and coupling with collective solvent modes. A future extension could combine the explicit first shell with point charges, QM/MM electrostatic embedding, polarizable embedding, or a continuum solvation model to approximate bulk effects. Previous PCM calculations for QMAP in DMSO showed that the average dynamic linear polarizability at 1064 nm increased by approximately 2% relative to the gas-phase value [3], indicating that dielectric embedding produces a measurable, although modest, effect on the molecular electronic response. Accordingly, embedding the present microsolvated cluster could refine the absolute values of the QMAP and cluster dipole moments and stabilize particular solvent orientations, whereas the principal short-range contact and hydrogen-bond motifs are expected to be less affected because the first-shell DMSO molecules are represented explicitly. Point-charge and continuum embeddings would, however, introduce additional assumptions concerning the external electrostatic field and solvent response. For this reason, the present study isolates the explicit first-shell contribution and treats a systematic comparison of bulk-embedding schemes as a subject for future simulations.

## Conclusion

This work used Car–Parrinello molecular dynamics to investigate solvent-induced polarization in an explicit first-shell microsolvation model composed of QMAP and DMSO. The main solvent effect observed here is not a large intrinsic change in the isolated QMAP dipole moment, but rather a change in the dipolar response of the microsolvated cluster associated with the reorganization of the first DMSO shell. The conformational rearrangement observed around 12–13 ps provides a structural link between the geometry of QMAP and the local electrostatic environment generated by the surrounding DMSO molecules.

The localized polarization model proposed in this study brings together, within a single interpretation, the moderate variation of the QMAP dipole moment, the larger dipole moment of the solvent-dominated cluster, the selective solute–solvent contacts in the phenolic region, and the absence of a persistent hydrogen-bond network. These results suggest that the first solvation shell contributes to the modulation of the local electrostatic environment of QMAP through directional and region-specific contacts. This behavior may not be fully captured by continuum solvent descriptions, in which local solvent organization is represented only through averaged dielectric effects.

The conclusions should, however, be interpreted within the limitations of the present microsolvated CPMD model. The analyzed trajectory length of approximately 32 ps and the finite number of DMSO molecules do not provide exhaustive sampling of all possible QMAP-DMSO configurations or bulk-solvent fluctuations. Therefore, the reorganization observed around 12–13 ps should be understood as a trajectory-dependent local rearrangement of the microsolvated cluster, rather than as a general transition time for QMAP in bulk DMSO. Longer trajectories and larger explicit-solvent models will be necessary to evaluate the transferability of these findings. Nevertheless, the present study supports a first-shell mechanism in which transient DMSO organization modulates the local electrostatic response of QMAP and should be further examined in extended simulations.

## Data Availability

The data generated and analyzed during the current study are available within the article and its Supplementary Information. Additional processed data, trajectory-analysis scripts, and related computational files are available from the corresponding author upon reasonable request.
